# Diterpenes from the Marine Algae of the Genus *Dictyota*

**DOI:** 10.3390/md16050159

**Published:** 2018-05-11

**Authors:** Jiayun Chen, Hong Li, Zishuo Zhao, Xue Xia, Bo Li, Jinrong Zhang, Xiaojun Yan

**Affiliations:** 1 Laboratory of Marine Natural Products, School of Marine Sciences, Ningbo University, Ningbo 315211, China; chenjiayun1030@163.com (J.C.); lih19921020@gmail.com (H.L.); zhaozishuo1102@163.com (Z.Z.); xiaxue980106@163.com (X.X.); lib980419@163.com (B.L.); 2 Key Laboratory of Applied Marine Biotechnology of Ministry of Education, Ningbo University, Ningbo 315211, China

**Keywords:** *Dictyota*, diterpene, secondary metabolites, bioactivity

## Abstract

Species of the brown algae of the genus *Dictyota* are rich sources of bioactive secondary metabolites with diverse structural features. Excellent progress has been made in the discovery of diterpenes possessing broad chemical defensive activities from this genus. Most of these diterpenes exhibit significant biological activities, such as antiviral, cytotoxic and chemical defensive activities. In the present review, we summarized diterpenes isolated from the brown algae of the genus.

## 1. Introduction

Marine brown algae of the genus *Dictyota*, belonging to the family Dictyotaceae, are mainly distributed in subtropical and tropical oceans [1]. Structurally diverse secondary metabolites from members of this genus were found to possess a defensive property which greatly contributes to their successful survival and reproduction in complex and diverse marine environments [2]. At present, hundreds of bioactive natural products, including terpenes, phenols [3], sterols [4], fatty acids [5], and polysaccharides [6], have been isolated from marine brown algae of the genus *Dictyota*. Diterpenes are a large class of structurally diverse natural products which are widely found in marine organisms, including *Dictyota* species [7]. Some diterpenes are promising drug candidates due to their remarkable pharmacological activity [8,9,10]. Some diterpenes from *Dictyota* species are considered as the characteristic constituents of this genus, and give them taxonomic significance [1,11]. Diterpenes from members of this genus usually exhibit potent cytotoxic or antiviral activities [12,13].

In the present review, we systematically summarize the structures and bioactivities of diterpenes derived from members of the genus *Dictyota*, with more than 80 references cited. Up to the end of 2017, a total of 233 diterpenes had been isolated from *Dictyota* species, most of which were from the marine brown alga *Dictyota dichotoma*. It has been reported that many of these diterpenes possess several interesting bioactivities, including cytotoxic and antiviral activities.

## 2. Diterpenes of Group I

Based on the revised biogenetic scheme widely cited, the diterpenes from *Dictyota* species can be divided into three groups (I–III), resulting from the first formal cyclization of the geranyl-geraniol precursor. Group 1 contains diterpenes derived by the first cyclization of the geranyl-geraniol precursor between C-1 and C-10 [1]. Diterpenes of Group 1 are mainly prenylated derivatives of known sesquiterpene skeletons, including prenylated-guaiane, prenylated-germacrane, and prenylated-*epi*-elemane. A total of 58 diterpenes of Group 1, including 47 prenylated-guaiane diterpenes, have been isolated from *Dictyota* species by the end of 2017. Most of the compounds exhibit biological properties, such as cytotoxic [14], antitumor [15], antiviral [16], antifouling [17] and antioxidant activities [15]. Table 1, Table 2 and Table 3 summarize 58 diterpenes of Group 1 derived from the *Dictyota* species (see in Section 2.1).

### 2.1. Prenylated-Guaiane Diterpenes

Up to the end of 2017, a total of 47 prenylated-guaiane diterpenes had been reported, and nearly half of them were isolated from *D. dichotoma*. Some prenylated-guaiane diterpenes from *Dictyota* species contain a chlorine substituent.

**Figure 1:**
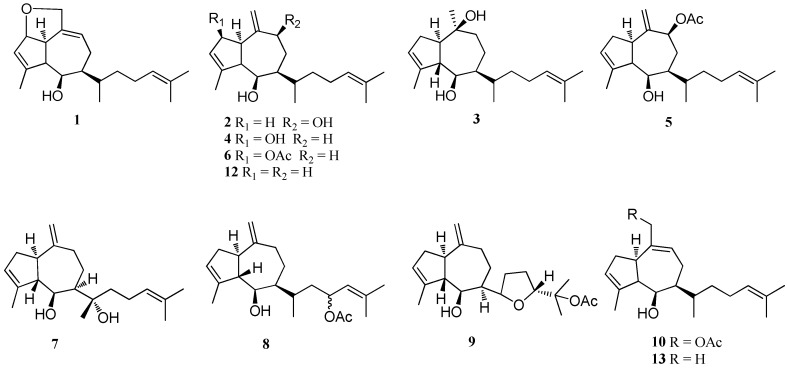

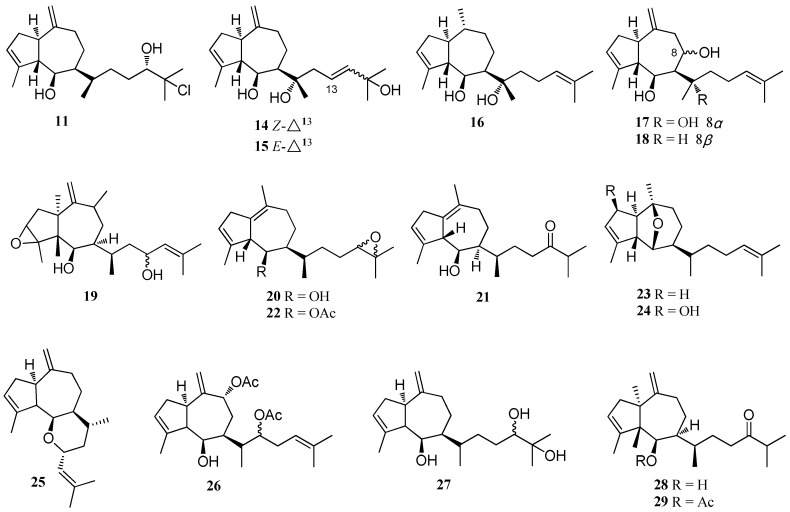
Chemical structures of **1**–**29**.

A family of cytotoxic diterpenes, named dictyols A–D (**1**–**4**) and dictyol B acetate (**5**), were isolated from *D. dichotoma* var. *implexa* which was collected from the Tyrrhenian Sea [18]. Compound **3** showed moderate antifouling activity against the freshwater mollusk *Limnoperna fortunei* without any toxic effects [17]. Compound **3** displayed weak protection activity against DNA damage, low antioxidant activity for ABTS (2,2′-azino-bis-3-ethylbenzthiazoline-6-sulfonic acid) and erythrocytes hemolysis [15]. Compound **5** exhibited moderate cytotoxic activity against human embryonic kidney cell line (Hek-293), oral carcinoma cells (KB), epithelial carcinoma of the larynx (Hep-2), breast cancer cells (MCF-7), and cervix adenocarcinoma (SiHa) cell lines with IC_50_ values ranging from 19.6 to 59.2 μg/mL. Compound **5** also showed weak antiproliferative activity against MCF-7 and SiHa cell lines with IC_50_ values of 38.3 and 34.4 μg /mL, respectively [14]. Compound **5** showed significant inhibition against the cyanobacterium *Oscillatoria perornata* with an IC_50_ value of 2.23 μM [19]. Additionally, **5** exhibited significant anti-herbivory activity against the crab *Pachygrapsus transversus* [20]. A novel diterpene, named dictyol-d-2*β*-acetate (**6**), was isolated from *D. dichotoma* collected near Puerto Madryn [21]. Dictyol E (**7**) was isolated from *D. dichotoma*, collected from the Red Sea coast of Egypt [22], and from several species of *Dictyota* in the Mediterranean region [23]. Compound **7** showed weak antibacterial activity against the marine bacterial strains *Pseudoalteromonas* sp. (D41), *Paracoccus* sp. (4M6) and *Polaribacter* sp. (TC5) with EC_50_ values of 100, 133, and 92 μM, respectively. Compound **7** also displayed a significant inhibitory effect on rat liver microsomal diacylglycerol acyltransferase with an IC_50_ value of 46.0 μM [24]. Dictyol G acetate (**8**) was obtained from *D. volubilis*, collected from the reef flat of Geoffrey Bay, Magnetic Island, Australia [25], and also from *D. binghamiae*, collected from Barkley Sound, British Columbia [26]. Dictyol H (**9**) was reported from *D. divaricata*, collected from the Great Barrier Reef region of Northern Australia [27], and also from *D. dentata* from the south west coast of Barbados [28]. Compound **9** displayed moderate antitumor activity against KB9 cell line with an IC_50_ value of 22 μg/mL [28]. Dictyol I acetate (**10**) was isolated from *D. dichotoma* var. *implexa* from the Northern Adriatic Sea [18]. A chlorine-containing diterpene, dictyol J (**11**), was reported from *D. dichotoma* by bioassay-guided isolation. Compound **11** exhibited high (more than 95%) algicidal activity against the red-tide phytoplankton *Heterosigma akashiwo* and *Karenia mikimotoi* at a dose of 10–20 μg/mL [29]. Prenylated-guaiane diterpenes, pachydictyol A (**12**) and isopachydictyol A (**13**) were isolated from several species of *Dictyota*, such as *D. menstrualis*, *D. caribaea*, *D. dichotoma* var. *Implexa*, and *D. volubilis* [14,15,17,18,20,30,31]. Compounds **12** and **13** showed potent antithrombotic effect through inhibition of thrombin, displaying an inhibition of 50% at 0.68 mM [30]. These compounds also displayed moderate to strong cytotoxicity against hepatoma (HepG2), fibroblast (WI-38), African green monkey kidney (VERO), and MCF-7 cell lines with IC_50_ values ranging from 22.4 to 40.2 μg/mL [15]. Compound **12** displayed a significant antifouling activity against the invading freshwater mussel *Limnoperna fortunei* at 4.7 μg cm^−2^ [17]. Three new diterpenes, named *cis*-pachydictyol B (**14**), *trans*-pachydictyol B (**15**), and pachydictyol C (**16**), were isolated from *D. dichotoma*, collected from the Red Sea coast of Egypt. Compounds **14** and **16** exhibited weak cytotoxicity against 12 human tumor cell lines with a mean IC_50_ value >30.0 μM. Compound **14** displayed a potent antimicrobial activity against the fungus *Mucor miehei*, and weak antifungal activity against *Candida albicans* and *Pythium ultimum* [22]. A new diterpene, named 8*α*,11-dihydroxypachydictyol A (**17**), was isolated from *D. plectens* [16] and from *Dictyota* sp., collected from Bang Saen Beach, Thailand [32]. Compound **17** displayed moderate antiviral activity against hemagglutinin-mediated viral entry with an inhibition rate of 56% at 30.0 μM [16]. Additionally, this compound also showed strong cytotoxicity against National Cancer Institute human small cell lung carcinoma (NCI-H187 cells) with an IC_50_ value of 5.0 μg/mL, and potent anti-malarial activity with an IC_50_ value of 3.22 μg/mL [32]. Another analog of pachydictyol A, 8*β*-hydroxypachydictyol A (**18**), was reported from *D. plectens* [16], *D. bartayresii* [33] and *D. dichotoma* var. *Implexa*, collected from the Red Sea [15]. This compound displayed weak cytotoxicity against HepG2, WI-38 (fibroblast cells), VERO and MCF-7 cell lines with IC_50_ values of 81.2, 62.6, 72.3, and 68.2 μg/mL, respectively [15]. Moreover, this compound was found to inhibit HIV-1 replication with an IC_50_ value of 26.1 ± 1.7 μM [16]. A new diterpene, named 3,4-epoxy-13-hydroxypachydictyol A (**19**), was obtained from *D. dichotoma*, collected in the Red Sea [34]. Three novel diterpenes, named acutilols A and B (**20** and **21**) and acutilol A acetate (**22**), were isolated from *D. acutiloba*, collected in Hawaii. Compounds **20**–**22** exhibited a significant feeding deterrent activity against both temperate and tropical herbivorous fishes as well as sea urchins [13,35]. Dictyoxide (**23**), isolated from Patagonian *D. dichotoma*, showed a potent antifouling activity against the invading freshwater mussel *L. fortune* at 4.7 μg cm^−2^ [17]. A prenylated-guaiane diterpene, named 2-hydroxydictyoxide (**24**), was isolated from *D. divaricata* from the Great Barrier Reef region of Northern Australia [27]. Two new diterpenes, dictyoxide A (**25**) and dictyotriol A diacetate (**26**), were identified from *D. binghamiae*, collected from Barkley Sound, British Columbia, while dictytriol (**27**) was isolated from a Japanese *D. dichotoma* [26]. Dictyone (**28**) and dictyone acetate (**29**) were isolated from *D. dichotoma* from the Red Sea coasts in Egypt [34]. Compound **28** and **29** showed moderate cytotoxicity against three proliferating mouse cell lines, a normal fibroblast line NIH3T3, and two virally transformed forms SSVNIH3T3 and KA3IT with IC_50_ values ranging from 5 to 35 μg/mL [36] (Figure 1).

**Figure 2:**
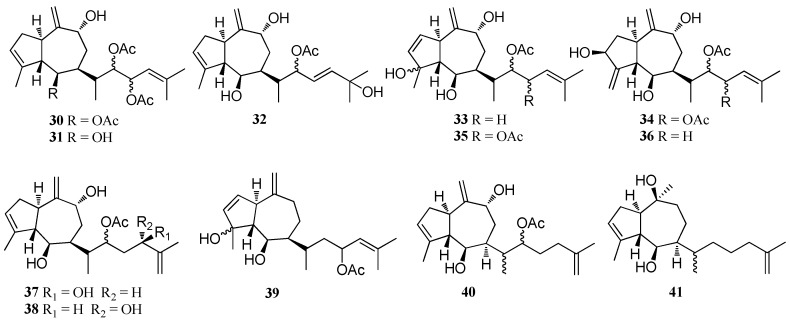

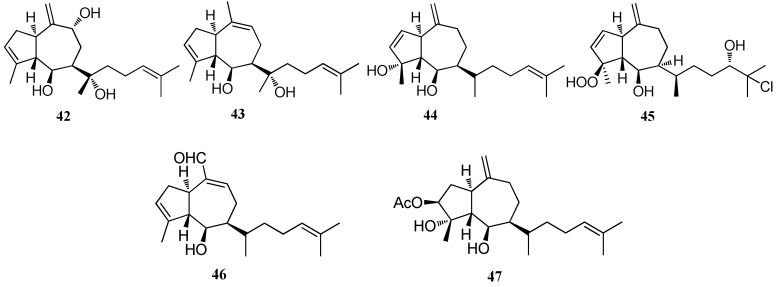
Chemical structures of **30**–**47**.

Compounds **30**–**33** were isolated from *D. volubilis* which was collected from Magnetic Island, Queensland, Australia [25]. Compounds **34**–**41**, which are highly oxidized prenylated-guaiane diterpenes, were reported from *D. volubilis* [31]. Two new diterpenes (**42** and **43**) were isolated from *D. plectens* which was collected from the South China Sea [16]. Dictyotadiol (**44**), isolated from Patagonian *D. dichotoma*, was found to display weak antifouling activity against the freshwater mollusk *L. fortunei* at 12 μg cm^−2^ [17]. Dictyohydroperoxide (**45**), a diterpene containing hydroperoxyl groups, was isolated from *D. dichotoma*, collected from the Troitsa Bay of Russian Far East. This compound was found to display a moderate cytotoxicity against HeLa, HL-60, and MDA-MB-231 human tumor cells and mouse epithelial cell line JB6 C141 with IC_50_ values of 71, 59, 201, and 68 μM, respectively [37]. A new diterpene, isopachydictyolal (**46**), was reported from *D. dichotoma* which was collected in the Saronicos Gulf in the Aegean Sea, Greece. This compound exhibited antiviral activity against Vero cells with a maximal non-toxic dose (MNTD) value of 10 μg/mL [38]. Compound **47** was obtained from the Mediterranean *Dictyota* spp. [23] (Figure 2).

### 2.2. Other Diterpenes of Group 1

**Figure 3:**
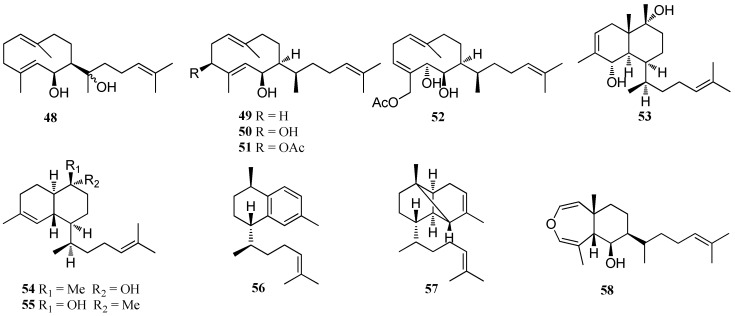
Chemical structures of **48**–**58**.

Besides prenylated-guaiane diterpenes, other diterpene skeletons belonging to Group 1 have also been isolated from members of *Dictyota* (Figure 3). A germacrane diterpene, named hydroxydilophol (**48**), was isolated from *D. masonii* which was collected at Isla Guadalupe in the Pacific of Mexico [39]. Two germacrane diterpenes (**49** and **50**) were isolated from *D. divaricata*, collected from the Great Barrier Reef region of Northern Australia [40]. Moreover, **50** was also isolated from the Mediterranean *Dictyota* sp. [41]. Two germacrane diterpenes, (**51** and **52**) were obtained from *D. plectens*, collected from the South China Sea. These compounds showed weak antiviral activity against HA-mediated viral entry at 30.0 μM [16]. Three cadinane diterpenes, named dictyotins A–C (**53**–**55**), were reported from *D. dichotoma* [42]. Two prenylated-cadinane diterpenes, **56** and **57**, were identified from *D. dichotoma*, collected from the Russian Far East [43], while **58**, a prenylated-epi-elemane diterpene, was isolated from *D. volubilis* [31].

## 3. Diterpenes of Group 1I

Based on the revised biogenetic scheme widely cited, Group II consists of diterpenes derived by a first cyclization of the geranyl-geraniol precursor between C-1 and C-11 [1]. The diterpene skeletons of this group comprise the dolabellane, dolastane, secodolastane etc. A total of 120 diterpenes of Group II, including 69 dolabellane diterpenes, were isolated from *Dictyota* species by the end of 2017, most of which exhibit biological properties, such as antibiotic [44], cytotoxic [45], antiviral [46], antibacterial [47], and protection activities against DNA damage [15] in addition to other biological activities. Table 4, Table 5 and Table 6 summarize 120 diterpenes of Group II identified from *Dictyota* species (see at the end of this section).

### 3.1. Dolabellane Diterpenes

Dolabellane diterpenes bearing the 5,11-fused bicyclic skeleton constitute a large number of diterpenes with structural diversity, including specific hydroxylation, oxidation, epoxidation, and other reactions [48]. A total of 69 compounds have been isolated from the genus *Dictyota*, among which 25 have been found from *D. dichotoma*. Dolabellane diterpenes were originally isolated from the opistobmnch mollusc *Dolabella californica* in 1977 [48]. Later, they were isolated from other marine organisms, including sponges, sea whips, and brown algae of the genus *Dictyota* [49].

**Figure 4:**
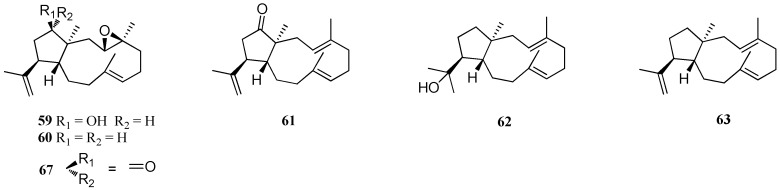

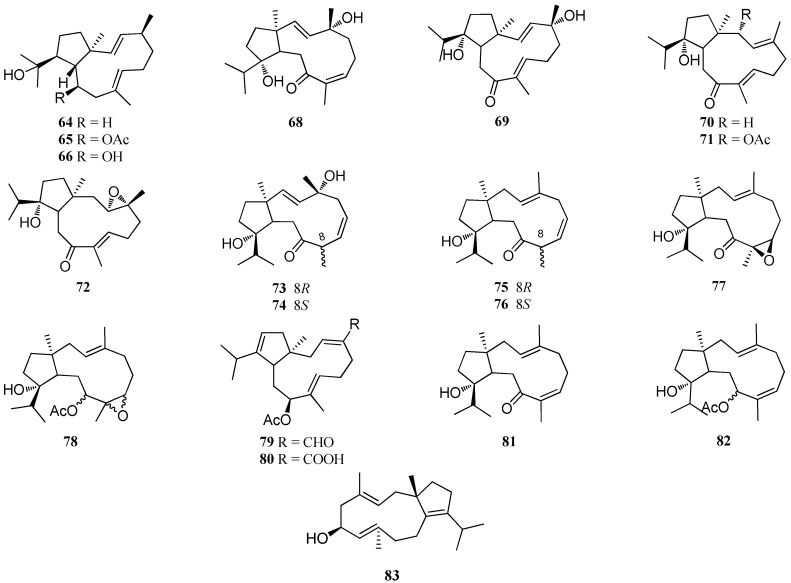
Chemical structures of **59**–**83**.

*D. dichotoma* is a chemically prolific member of the genus *Dictyota* since there are 25 structurally diverse dolabellane diterpenes from this alga (Figure 4). Nine dolabellane diterpenes, **59**–**67**, were isolated from *D. dichotoma*, collected from Acicastello near Catania, Sicily, Italy [44]. Compound **67** showed strong cytotoxic activity against murine leukemia cells (P-388), human nasopharynx carcinoma (KB) and human non-small cell lung carcinoma (NSCLCN6-L16) cells with ED_50_ values of 6.5, 25.39, and 16.66–16.78 μg/mL, respectively [50]. Fifteen novel dolabellanes, **68**–**82**, were reported from *D. dichotoma* collected in Krusadai Island, Gulf of Mannar, India in 1983 [51]. In addition, **79**–**82** were isolated from *D. bartayresiana*, collected on the coast of Hare Island, Gulf of Mannar, India in 1985 [52]. A cytotoxic diterpene, named dolabellatrienol (**83**), was isolated from the Red Sea *D. dichotoma* var. *implexa*, and it showed moderate in vitro cytotoxicity against four human tumor cell lines, HepG2, WI-38, VERO, and MCF-7, with IC_50_ values of 102.3, 100.6, 120.6, and 150.5 μg/mL, respectively [15].

**Figure 5:**
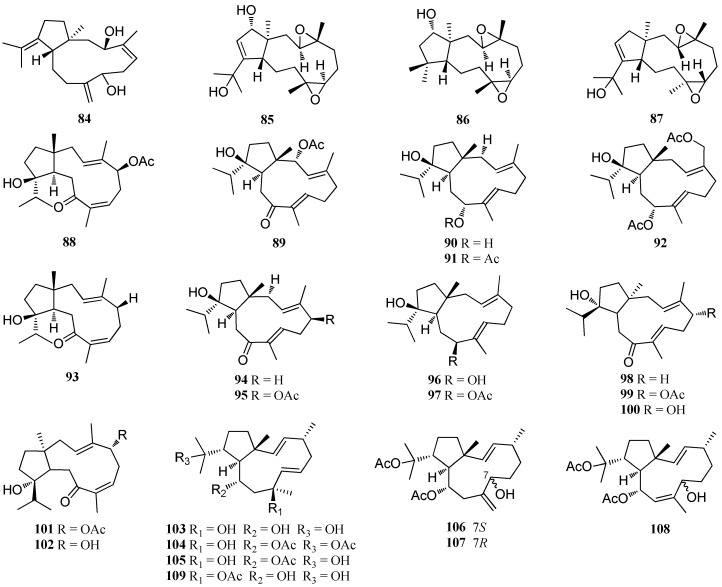

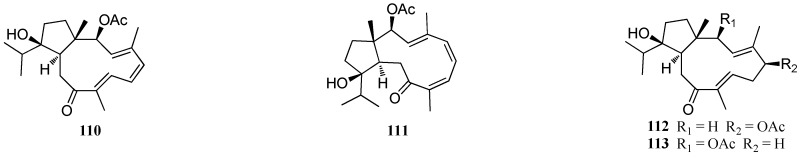
Chemical structures of **84**–**113**.

Besides *D. dichotoma*, other algae of this genus are also rich producers of bioactive dolabellane diterpenes (Figure 5). Fifteen compounds **84**–**98** were isolated from *D. pardarlis* f. *pseudohamata* from Magnetic Island [53,54,55]. Compounds **98**–**102** were also reported from *D. bartayresiana*, collected from Hare Island in the Gulf of Mannar of the Indian Ocean [52]. Three dolabellane diterpenes, **103**–**105**, were isolated from *D. pfaffii*. Three antiviral diterpenes, named dolabelladienols A–C (**106**–**108**), were found from the same species, collected from Atol das Rocas, Northeast Brazil [46]. Compound **103** showed potent anti-HIV-1 effect ranging from 60% to 90% in peripheral blood cells (PBMC) and macrophages infected with the human immunodeficiency virus (HIV) from 60% to 90%, respectively [56]. This compound also exhibited moderate inhibition against herpes virus at a concentration of 50 μM, and it was found to be moderately active against HIV-1 reverse transcriptase activity at a concentration of 40 μM [57]. Moreover, **103** also displayed significant antimalarial activity against *Leishmania amazonensis* with an IC_50_ value of 44 μM [58]. Compound **104**, an antifeedant against the sea urchin and generalist fishes [13], exhibited strong anti-HSV-1 activity with a CC_50_ value of 185 ± 5 μM [57]. Compounds **106** and **107** exhibited strong anti-HIV-1 activity with IC_50_ values of 2.9 and 4.1 μM, respectively [46]. Compound **109** was isolated from *D. pfaffii* which was collected from Atol das Rocas in Northeast Brazil, and it displayed strong anti-HSV-1 activity, reaching an inhibition of 87% at a concentrate of 50 μM [57]. Four antiviral diterpenes, **110**–**113**, have been extracted from *D. plectens* which was collected from the South China Sea. These compounds showed specific inhibition against HA-mediated viral entry with an inhibition rate of 62% at 30.0 μM [16].

**Figure 6:**
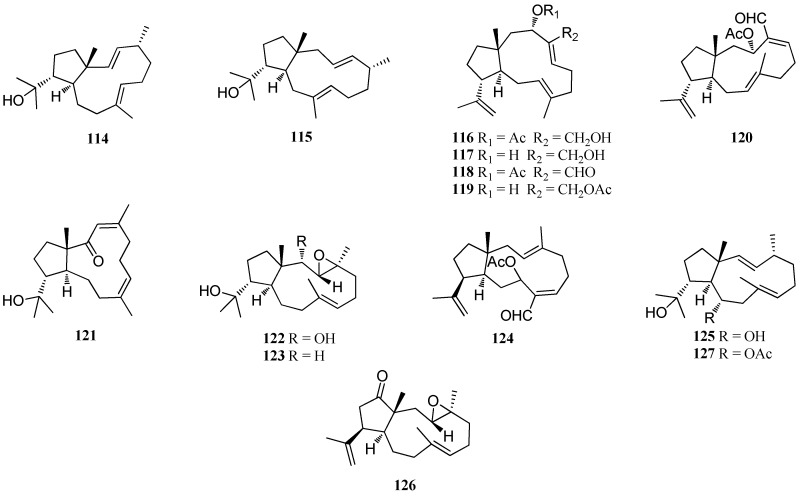
Chemical structures of **114**–**127**.

Besides the above-mentioned algae, other member of the genus *Dictyota* are also producers of bioactive dolabellane diterpenes (Figure 6). Compound **114** was isolated from *D. divaricata* collected from the Great Barrier Reef region of Northern Australia [40]. Compound **115** was isolated from *D. volubilis* [31]. On the other hand, **116**–**120** were isolated from *Dictyota* sp., collected near Portopalo. Compound **116** displayed significant in vitro cytotoxicity against KB cells [45]. Three antifouling compounds **121**–**123**, were obtained during an investigation of a Mediterranean *Dictyota* sp. Compound **122** showed moderate antifouling activity against marine bacterial biofilm-forming bacteria D41 with an EC_50_ value of 110 μM, while compound **123** was weakly active with an EC_50_ value of 250 μM [41]. Four antifouling compounds **124**–**127** were isolated from *Dictyota* spp. collected from the Mediterranean coasts (France and Algeria) [23]. Both compounds **126** and **127** displayed weak anti-adhesion activity against D41 with an EC_50_ more than 100 μM. These compounds showed weak antibacterial activity against macrolide-resistant variant RN4220 with MIC values of 128 and 64 μg/mL, respectively [59]. Moreover, compound **127** exhibited selective inhibitory activity against the cyanobacterium *Oscillatoria perornata* with an IC_50_ value of 23.4 μM [19].

### 3.2. Dolastane Diterpenes

Dolastane diterpenes containing the 5,7,6-tricyclic skeleton are another class of bioactive constituents of brown algal species of the genus *Dictyota* [60]. At present, a total of 38 dolastane diterpenes have been obtained from *Dictyota* species.

**Figure 7:**
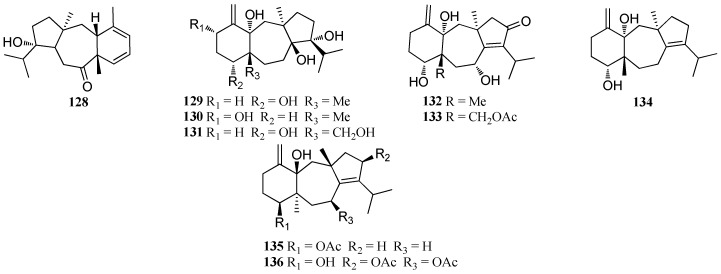
Chemical structures of **128**–**136**.

Compound **128** was isolated from *D. dichotoma*, collected from the coast of the Indian Ocean [51], and also from *D. bartayresiana*, collected in the Gulf of Mannar of the Indian Ocean [52] while **129** was isolated from *D. cervicornis* [61] and *D. dichotoma* [62]. Two dolastane diterpenes, dichototetraol (**130**) and dichotopentaol (**131**), were isolated from *D. dichotoma*, collected from the Karachi Coast of the Arabian Sea [62]. Two diterpenes, named dichotenone A (**132**) and dichotenone B (**133**), were reported from the marine alga *D. dichotoma* [47], while amijiol (**134**) was isolated from *D. indica*, collected from Bulegi near the Karachi Coast of the Arabian Sea [63]. Compound **134** showed moderate antitumor activity [15]. Extensive efforts to discover bioactive natural products from the Red Sea *D. dichotoma* var. *Implexa* resulted in the isolation of three cytotoxic diterpenoids, amijiol (**134**), amijiol acetate (**135**), and amijiol-7,10-diacetate (**136**). Compound **135** exhibited strong antitumor activity against HepG2, WI-38, VERO, and MCF-7 with IC_50_ values of 25.1, 14.2, 20.5, and 21.2 μg/mL, while **136** gave IC_50_ values of 47.0, 16.2, 21.4 and 30.5 μg/mL, respectively. Moreover, both **135** and **136** displayed potent anti-oxidative activity [15] (Figure 7).

A bioactive diterpene **137** was isolated from a Brazilian *D. cervicornis* [64]. This compound showed a strong antimalarial activity against promastigote, axenic amastigote and intracellular amastigote forms of *Leishmania amazonensis* with IC_50_ values of 2.0, 12.0, and 4.0 μg/mL, respectively [65], in addition to antifouling effect [66] and inhibitory activity against the mammalian Na^+^K^+^-ATPase [64]. Compounds **138**–**140** were obtained from *D. cervicornis*, collected from Baia da Ribeira, Brazil [61]. Compound **141**, was isolated from a Brazilian *D. cervicornis* [64] and was found to inhibit strong antifeedant activity with a herbivory inhibitory effect (HIE) value of 70% [67] as well as antifouling activity against the mussel *Perna perna* [66]. Moreover, this compound also displayed significant inhibitory effect on HIV-1 replication with an EC_50_ value of 0.3 μM [68].

**Figure 8:**
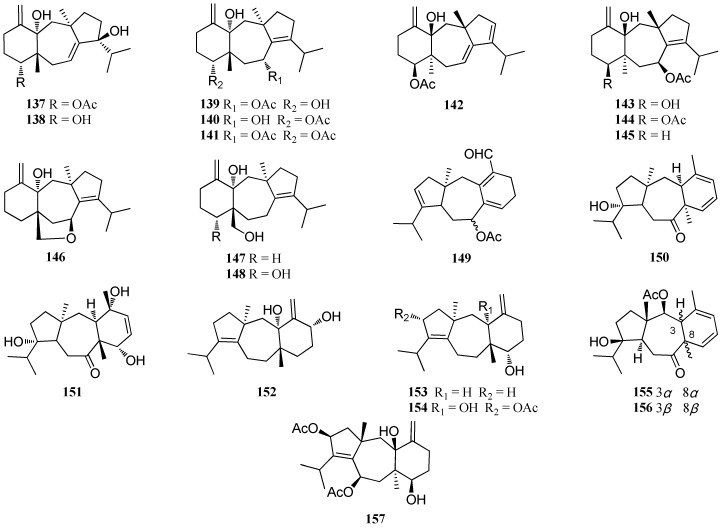
Chemical structures of **137**–**157**.

Four dolastane diterpenes, **142**–**145**, were isolated from *D. divaricata*, collected from the Virgin Islands [69]. Examination of the organic extract of *D. indica*, collected from Bulegi near the Karachi Coast of the Arabian Sea provided three diterpenes, dictinol (**146**), dictindiol (**147**), and dictintriol (**148**) [63]. Compounds **149**–**151** were reported from *D. bartayresiana*, collected in the Hare Island of the Gulf of Mannar of the Indian Ocean [52]. Three dolastane diterpenes, named isoamijiol (**152**), 14-deoxyamijiol (**153**), and amijidictyol (**154**), were isolated from *D. linearis* [70] and a total synthesis of compound **152** was accomplished [71]. Compounds **155**–**157** were isolated from *D. plectens*, collected from the South China Sea and were found to exhibit a weak anti-inflammatory activity against lipopolysaccharide (LPS)-induced nitric oxide (NO) production at 10.0 μM [16] (Figure 8).

**Figure 9:**
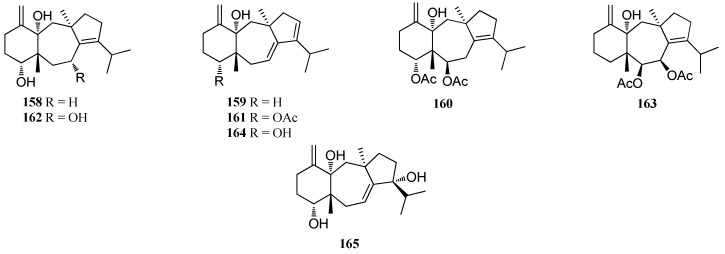
Chemical structures of **158**–**165**.

Extracts of the mixed collections of two brown algae *D. linearis* and *D. divaricata*, from the Honduras Bay Islands, afforded seven dolastane diterpenes **137**, **138**, and **158**–**162**. Compound **161** displayed a strong reversible inhibitory action of histamine on the guinea pig ileum at a concentration of 16 μg/mL. Compound **162** showed moderate decrease in the twitch height of rat hemidiaphragm preparation at a concentration of 16 μg/mL. Moreover, **162** displayed weak inhibition of cell division using an urchin egg assay [60]. Compound **163** was isolated from *D. furcellata*, collected from Cape Peron in Western Australia [72]. Two dolostane diterpenes **164** and **165**, were isolated from *Dictyota* sp. from the Canary Islands [73] (Figure 9).

### 3.3. Secodolastane Diterpenes

**Figure 10:**
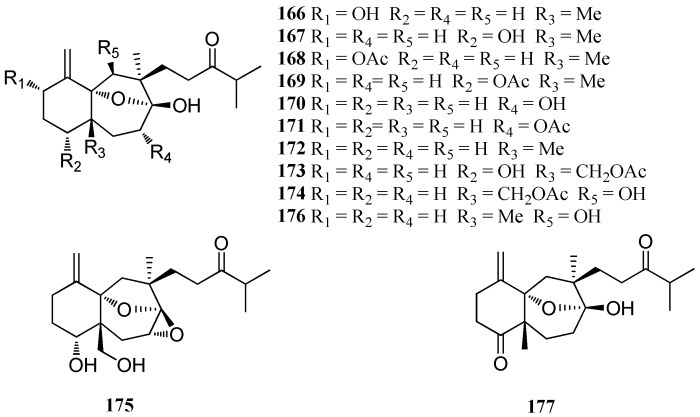
Chemical structures of **166**–**177**.

Secodolastane diterpenes are a class of compounds derived by decyclization of the dolastane skeleton between C-8 and C-9 [74]. A total of 12 secodolastane diterpenes were found in *D. indica*, *D. cervicornis*, or *D. dichotoma* (Figure 10).

Six secodolastane diterpenes, named linearol (**166**), isolinearol (**167**), linearol acetate (**168**), isolinearol acetate (**169**), cervicol (**170**), and cervicol acetate (**171**), were isolated from *D. cervicornis*, collected from Baia da Ribeira, Brazil [74]. The extracts of *D. indica* of the Arabian Sea furnished linearol (**166**), isolinearol (**167**), indicol (**172**), and indicarol acetate (**173**) [75]. Four secodolastane diterpenes, named dichotenols B and C (**174** and **175**), dichotone (**176**), and dichotodione (**177**), were isolated from *D. dichotoma*. Both **174** and **176** exhibited significant antibacterial and antifungal activities [47].

### 3.4. Dictyoxetane Diterpenes

**Figure 11:**
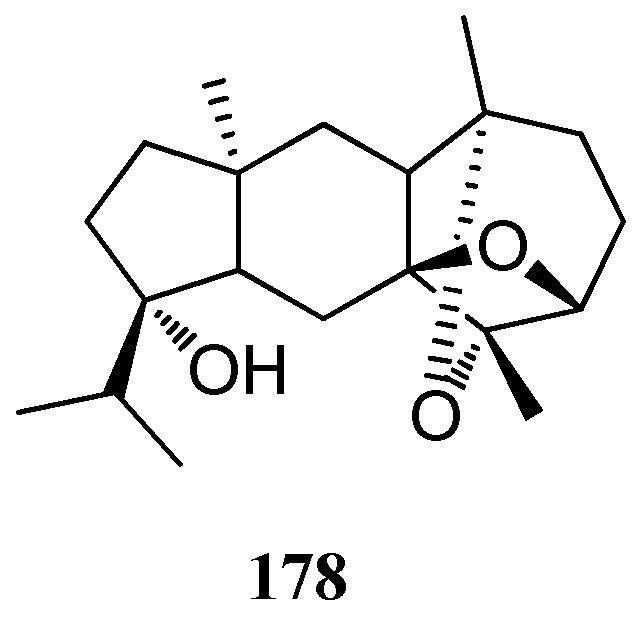
Chemical structure of **178.**

A dictyoxetane diterpene **178** was isolated from *D. dichotoma*, collected from the coast of the Indian Ocean [51] (Figure 11).

## 4. Diterpenes of Group III

The diterpenes of this group are derived from cyclization of the geranyl-geraniol precursor between C-2 and C-10 or by ring contraction of the prenylated-germacrane [1]. Xenicane diterpenes, the main diterpenes of Group III, undergo oxidation, epoxidation, condensation, and other reactions to give rise to monocyclic, bicyclic, and tricyclic structures. Forty xenicane diterpenes were isolated from members of the genus *Dictyota* and most of them exhibited interesting biological activities, such as antiviral [16], anti-inflammatory [76], cytotoxic [12], antifungal [77], and other biological activities. Table 7 and Table 8 summarize 55 diterpenes of Group III from *Dictyota* species (see in Section 4.1).

### 4.1. Xenicane Diterpenes

**Figure 12:**
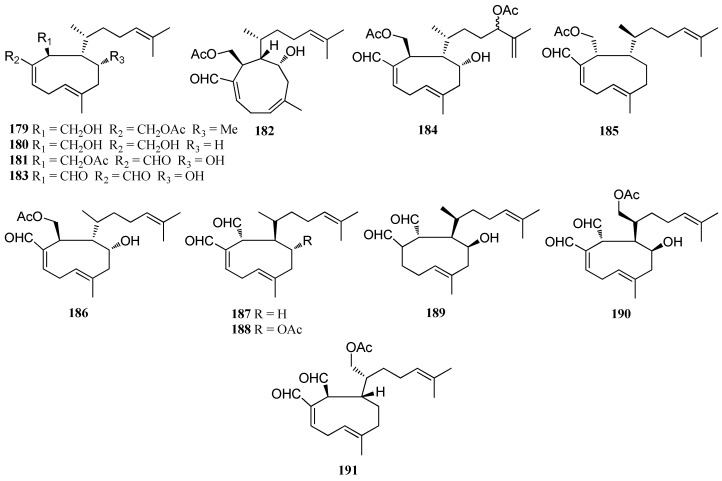
Chemical structures of **179**–**191**.

Xenicane diterpenes are a large class of marine diterpenes bearing a cyclononane ring as a common structural feature. The species of the genus *Dictyota* have been shown to be important producers of xenicane diterpenes since 40 xenicanes were isolated from members of this genus. Antiviral compounds, **179**–**183**, were obtained from *D. plectens* from the South China Sea. Compound **181** showed moderate inhibition against HIV-1 replication with an IC_50_ value of 21.9 ± 1.3 μM. Compound **183** displayed moderate antiviral activity against HA-mediated viral entry and strong anti-inflammatory activity against LPS-induced NO production at 10.0 μM [16]. Compound **184** was isolated from *D. plectens*, collected from the Xuwen coast, China and was found to exhibit a weak anti-inflammatory activity against LPS-induced NO production at 10.0 μM [76]. Two cytotoxic diterpenes, acetyldictyolal (**185**) and hydroxyacetyldictyolal (**186**), were isolated from *D. dichotoma*, collected at Oshoro Bay, Hokkaido [78]. Compound **185** displayed strong cytotoxicity against P-388, KB, NSCLCN6-L16 cell lines with EC_50_ values ranging from 1.50 to 9.1 μg/mL and weak antifungal activity against *Aspergillus fumigates* (IPC864-64), *Microsporum canis* (IPC1687-87) and *Trichophyton mentagrophytes* (IPC1468-83) [50]. Dictyodial (**187**) and 4*α*-acetyldictyodial (**188**) were isolated from *D. linearis*, collected from the south coasts of Chios Island [38]. Compound **187** was also isolated from *D. crenulata* and *D. flabellata*, respectively. Compound **187** exhibited potent antibacterial activity against *Staphylococcus aureus* and *Bacillus subtilis* as well as antifungal activity against *C. albicans* [77]. Hydroxydictyodial (**189**), isolated from *D. spinulosa* collected from Kin, Okinawa was found to exhibit a potent antifeedant activity against the omnivorous fish *Tilapia mossambica* as well as antibiotic activity against *S. aureus* and *B. subtilis* [79]. Compound **190** was reported from *D. divaricata* from the Great Barrier Reef region of Northern Australia [27]. 17-Acetoxy-dictyodial (**191**), isolated from *D. ciliolata* collected from the Oualidia lagoon was found to exhibit moderate antifungal activity against *C. albicans* with MIC value of 50 μg/mL [80] (Figure 12).

**Figure 13:**
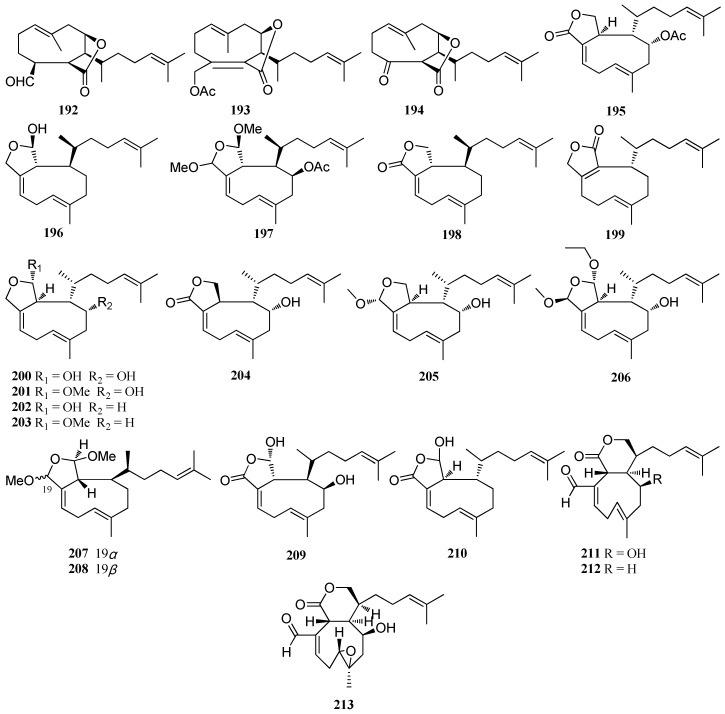
Chemical structures of **192**–**213**.

Four cytotoxic diterpenes, dictyotalide A (**192**), dictyotalide B (**193**), nordictyotalide (**194**), and 4-acetoxydictyolactone (**195**), isolated from *D. dichotoma* which was collected at Yagachi, Okinawa, exhibited significant cytotoxic activity against mouse melanoma cells (B16) with IC_50_ values of 2.57, 0.58, 1.58, and 1.57 μg/mL, respectively [12]. Isodictyohemiacetal (**196**) and dictyodiacetal (**197**) were isolated from *D. dichotoma*, collected from Oshoro Bay, Hokkaido [78]. A rare algicidal diterpene, named dictyolactone (**198**), was reported from *D. dichotoma*. This compound showed high algicidal activity against representative harmful algal bloom (HAB) species *Heterosigma akashiwo* and *Karenia mikimotoi*, and moderate insecticidal activity against the dinoflagellate *Alexandrium catenella* [29]. Neodictyolactone (**199**) was isolated from *D. linearis* from the south coasts of Chios Island [38]. Both **198** and **199** displayed a weak antifungal activity against the fungal strains IPC864-64, IPC1687-87, and IPC1468-83 as well as excellent cytotoxicity against NSCLCN6-L16 cells with EC_50_ values of 0.3 and 2.0 μg/mL, respectively. Moreover, **198** showed significant cytotoxicity against P-388 cells, P-388/DOX cells, and KB cells with EC_50_ values of 2.8, 2.4, and 4.9 μg/mL, while **199** was less active with EC_50_ values of 3.4, 3.9, and 6.2 μg/mL, respectively [50]. Compounds **200**–**205** were isolated from *D. plectens* collected from the Xuwen coast. Compounds **200** and **205** showed an inhibitory effect on the replication of a wild-type HIV-1 with IC_50_ values of 28.1 and 25.4 μM, respectively while **204** displayed moderate antiviral activity against HA-mediated viral entry with an inhibition rate of 66.8% at a concentration of 30.0 μM. Moreover, **204** exhibited significant anti-inflammatory effect by inhibiting LPS-induced NO production with an inhibition rate of 76.0% at a concentration of 10.0 μM [76]. Compound **206** was isolated from *D. plectens* from the South China Sea. Compound **206** showed weak antiviral activity against HA-mediated viral entry [16]. Compounds **207** and **208** were reported from *Dictyota* sp. collected from Bahia de Los Angeles [81]. A rare anti-tuberculosis diterpene **209** was isolated from from *Dictyota* sp. collected from the Bang Saen Beach, Thailand and was found to display a weak anti-tuberculosis activity against *Mycobacterium tuberculosis* with an MIC value of 200 μg/mL [32]. Compound **210** was reported from Mediterranean *Dictyota* spp. collected from the Mediterranean coasts of Algeria [23]. Three novel xenicane diterpenes, compounds **211**–**213**, were isolated from *D. divaricata* collected from the Great Barrier Reef region of Northern Australia [27] (Figure 13).

**Figure 14:**
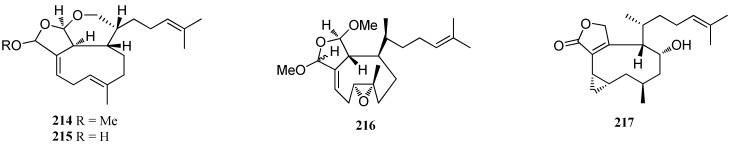
Chemical structures of compounds **214**–**217**.

A tricyclediterpene **214** was isolated from *D. divaricata* collected from the Great Barrier Reef region of Northern Australia [40] while ciliolatale (**215**) was isolated from *D. ciliolata* from the Oualidia lagoon [80]. A bioactive diterpene, named dictyoepoxide (**216**), was isolated from *Dictyota* sp. collected in Bahia de Los Angeles and was found to exhibit a high vasopressin receptor antagonist activity in vitro [81]. A tricyclediterpene, named 4*α*-hydroxycrenulatane (**217**) was obtained from *Dictyota* sp. collected from Bang Saen Beach, Thailand [32] (Figure 14).

**Figure 15:**
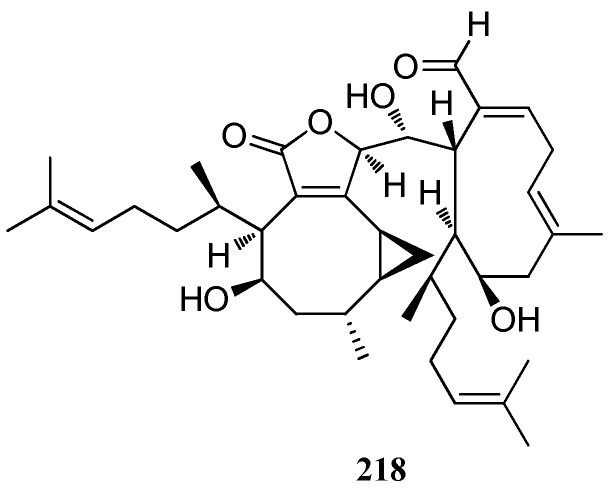
Chemical structure of **218**.

An unusual dissymmetrical dimer, dictyotadimer A (**218**) which contains two different xenicane units, was isolated from a Mediterranean brown seaweed *Dictyota* sp. Compound **218** is the first diterpene dimer of algal origin and a plausible biogenetic pathway of compound **218** has been proposed [82] (Figure 15).

### 4.2. Crenulidane Diterpenes

**Figure 16:**
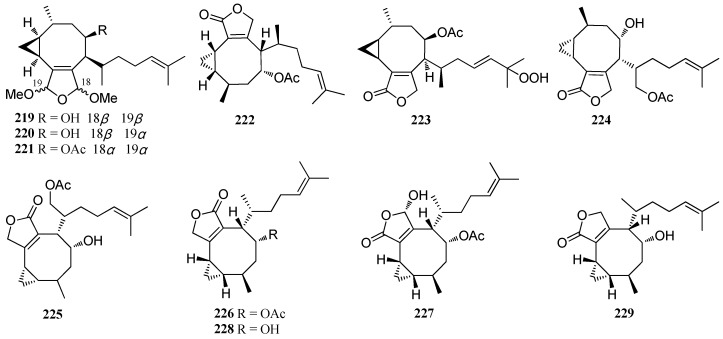
Chemical structures of **219**–**229**.

Two crenulidane diterpenes, named crenulacetal A (**219**) and crenulacetal B (**220**), were isolated from *D. spinulosa* [83]. Moreover **219**, together with crenulacetal C (**221**), were also isolated from *D. dichotoma* [83,84]. Compound **221** displayed significant pesticidal activity against the larvae of *Polydora websterii* at a concentration of 1.5 ppm [84]. A piscicidal diterpene, named acetoxycrenulide (**222**), was isolated from *D. dichotoma* [37], and from Mediterranean *Dictyota* spp. [23]. Compound **222** exhibited strong fish antifeedant activity due to its piscicidal activity [83]. Compound **222** also displayed an anti-microfouling activity against three marine bacterial strains D41, 4M6, and TC5 with EC_50_ values of 82 ± 28, 69 ± 17, and 154 ± 20 μM, respectively [23]. Moreover, a total synthesis of **222** has been accomplished [85]. Compound **223** was isolated from *D. dichotoma* collected in Troitsa Bay of the Peter the Great Bay [37]. Two crenulidanes **224** and **225** were isolated from *D. divaricata* collected from the Great Barrier Reef region of Northern Australia, [27,40] while **226**–**228** were reported from *D. plectens*. Compounds **226** and **227** displayed weak antiviral activity by inhibition of HA-mediated viral entry at 30.0 μM [16,76] while **228** showed a moderate anti-inflammatory effect by inhibiting LPS-induced NO production with an inhibition rate of 53.2% at a concentration of 10 μM [76]. Hydroxycrenulide (**229**) was isolated from a Mediterranean *Dictyota* sp. Compound **229** showed weak antifouling activity against the marine bacterial strain D41 [41] (Figure 16).

### 4.3. Dichotomane Diterpenes

**Figure 17:**
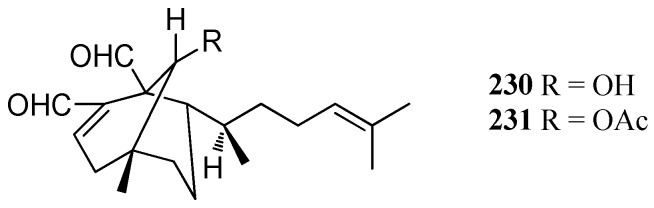
Chemical structures of **230**–**231**.

Two antiviral diterpenes, named Da-1 (**230**) and AcDa-1 (**231**), were isolated from *D. menstrualis* collected from Brazil. Compounds **230** and **231** exhibited significant antiretroviral activity against HIV-1 replication with EC_50_ values of 40 and 70 μM, respectively [86]. Compound **230** was also isolated from *D. pfaffii* and exhibited inhibitory activity against HSV-1 replication with an EC_50_ value of 5.10 μM [87]. Additionally, **230** was found to display other bioactivities, including thrombin inhibition [30], anti-feeding activity [88], and herbicide activity against pasture weeds [89] (Figure 17).

### 4.4. Crenulane Diterpenes

**Figure 18:**
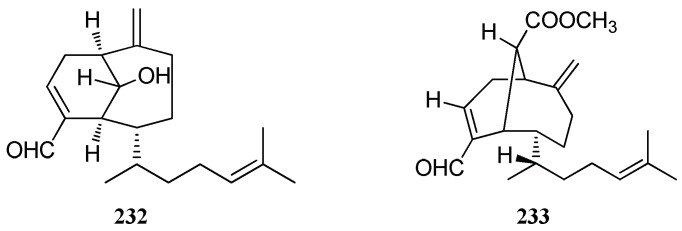
Chemical structures of **232**–**233**.

An antialgal diterpene, named sanadaol (**232**), was isolated from *D. dichotoma*. Compound **232** showed high antialgal activity (>95%) against the red-tide phytoplankton *H. akashiwo* and *K. mikimotoi* at a dose of 10–20 μg/mL [29]. Another crenulane diterpene, acetylsanadaol (**233**), was identified from *D. linearis* from the south coasts of Chios Island [38] (Figure 18).

## 5. Conclusions

The genus *Dictyota* is a rich source of various natural products with unprecedented pharmacological and biological activities. Significant progress has been made in the discovery of bioactive secondary metabolites from members of the genus *Dictyota* [90]. The overwhelming majority of those secondary metabolites are diterpenes, especially Group II diterpenes (120 compounds) accounting for almost half of the total diterpenes from the *Dictyota* species (233 compounds). The cosmopolite *D. dichotoma*, the species that produces diterpenes of all three groups (I–III), has been proven to be an important producer of diterpenes. A total of 78 structurally diverse diterpenes have been isolated from *D. dichotoma*.

Some diterpene skeletons from *Dictyota* species are the characteristic constituents of this genus, which have chemotaxonomic significance. For example, the majority of prenylated-guaiane and dolabellane diterpenes were isolated from *D. dichotoma*, while dolastane diterpenes are mainly found in three species *D. dichotoma*, *D. divaricato*, and *D. linearis*. Xenicane diterpenes, a class of chemical characteristic for the taxonomy of the genus *Dictyota*, are found in only a few *Dictyota* species, mainly in *D. plectens*.

However, there are a number of problems in drug discovery and development from *Dictyota* species, including the development of new techniques applied to discover more bioactive diterpenes, total synthesis, multi-target screening assay, and pharmacological mechanisms of drug candidates. Firstly, it is necessary to discover more bioactive secondary metabolites from *Dictyota* species using a combined multi-target screening assay, bioassay-guided separation with an LC–MS based metabolomics approach in further research. Secondly, few results have been achieved in the total synthesis of bioactive diterpenes. The total synthesis of compounds **152** and **222** has been successfully completed. More efforts should be devoted in improving the total synthesis of bioactive diterpenes from the genus *Dictyota*. Successful total synthesis would be beneficial for the structural optimization of natural diterpenes, for further biological activity evaluation, and for pharmacological and clinical applications. Thirdly, as for bioactivity evaluation, less than half of the diterpenes derived from the *Dictyota* species have been measured due to the limitations of bioactivity assays. Various biological activity assays, including multi-target screening assay, in vitro and animal experiments, should be improved to promote the discovery of new promising leader drugs.

This review summarized diterpenes derived from the genus *Dictyota* up to the end of 2017, providing valuable insight into the further discoveries of novel diterpenes from the genus *Dictyota*.

## Figures and Tables

**Table 1:** Bioactivities of prenylated-guaiane diterpenes (**1**–**29**) from the genus *Dictyota*.

Structure Class	Metabolites	Sources	Activities	References
Dictyols	Dictyols A and B (**1**, **2**)	*D. dichotoma* var. *implexa*, Tyrrhenian sea	nd (not determined)	[18]
	Dictyol C (**3**)	*D. divaricata*, Great Barrier Reef region *D. dentata*, Boomers Beach Barbados *D. dichotoma* var. *implexa*, Tyrrhenian sea *D. dichotoma*, Patagonia	Protection for DNA damage; Antitumor activity; Antioxidant activity; Antifouling activity	[15,17,18,27,28]
	Dictyol D (**4**)	*D. dichotoma* var. *implexa*, Tyrrhenian sea	nd	[18]
	Dictyol B acetate (**5**)	*D. dichotoma* var. *Implexa*, Tyrrhenian sea *D. caribaea*, *Dictyota ciliolata*, Caribbean coast, Yucatan peninsula	Significant anti-herbivory activity; Selective antialgal activity; Moderate cytotoxicity; Antiproliferative activity	[14,18,19,20]
	Dictyol-d-2*β*-acetate (**6**)	*D. dichotoma*, near Puerto Madryn	nd	[21]
	Dictyol E (**7**)	*D. dichotoma*, Red Sea, Egypt *Dictyota* spp., Mediterranean Sea	Weak antimicrobial property; Moderate diacylglycerol acyltransferase inhibitory activity	[22,23,24]
	Dictyol G acetate (**8**)	*D. volubilis*, Geoffrey Bay, Australia *D. binghamiae*, Barkley Sound, British Columbia	nd	[25,26]
	Dictyol H (**9**)	*D. divaricata*, Great Barrier Reef region *D. dentata*, Boomers Beach	Moderate antitumor activity	[27,28]
	Dictyol I acetate (**10**)	*D. dichotoma* var. *Implexa*, Northern Adriatic sea	nd	[18]
	Dictyol J (**11**)	*D. dichotoma*	High algicidal activity	[29]
Pachydictyols	Pachydictyol A (**12**)	*D. dichotoma* var. *Implexa*, Northern Adriatic Sea *D. menstrualis*, Brazil *D. dichotoma*, Patagonia *D. caribaea* *D. ciliolata*, Caribbean coast, Yucatan peninsula *D. dichotoma* var. *implexa*, Red Sea *D. volubilis*	Potent antithrombotic activity; Moderate cytotoxicity; Potent antifouling activity	[14,15,17,18,20,30,31]
	Isopachydictyol A (**13**)	*D. menstrualis*, Brazil *D. caribaea* *D. dichotoma* var. *implexa*, Red Sea	Potent antithrombotic activity; Strong cytotoxicity	[15,20,30]
	*Cis*-pachydictyol B (**14**)	*D. dichotoma*, Red Sea, Egypt	Potent antimicrobial property; Weak cytotoxicity	[22]
	*Trans*-pachydictyol B (**15**)	*D. dichotoma*, Red Sea, Egypt	nd	[22]
	Pachydictyol C (**16**)	*D. dichotoma*, Red Sea, Egypt	Weak cytotoxicity	[22]
	8*α*,11-Dihydroxy- pachydictyol A (**17**)	*Dictyota* sp., Bangsaen Beach, Thailand *D. plectens*, South China Sea	Strong cytotoxicity; Potent anti-malarial activity; Antiviral activity	[16,32]
	8*β*-Hydroxy- pachydictyol A (**18**)	*D. dichotoma* var. *implexa*, Red Sea *D. bartayresii*, Geoffrey Bay, Australia *D. plectens*, South China Sea	Weak cytotoxicity; Antiviral activity	[15,16,33]
	3,4-Epoxy-13-hydroxy-pachydictyol A (**19**)	*D. dichotoma*, Red Sea, Egypt	nd	[34]
Acutilols	Acutilols A and B (**20**, **21**) Acutilol A acetate (**22**)	*D. acutiloba*, Tunnels Beach, Hawaii	Potent feeding deterrent	[13,35]
Dictyoxides	Dictyoxide (**23**)	*D. dichotoma*, Patagonia	Potent antifouling activity	[17]
	2-Hydroxydictyoxide (**24**)	*D. divaricata*, Great Barrier Reef region	nd	[27]
	Dictyoxide A (**25**)	*D. binghamiae*, Barkley Sound, British Columbia	nd	[26]
Dictytriols	Dictyotriol A diacetate (**26**)	*D. binghamiae*, Barkley Sound, British Columbia	nd	[26]
	Dictytriol (**27**)	*D. dichotoma*, Japan	nd	[26]
Dictyones	Dictyone (**28**) Dictyone acetate (**29**)	*D. dichotoma*, Red Sea, Egypt	Moderate cytotoxicity	[34,36]

**Table 2:** Bioactivities of prenylated-guaiane diterpenes (**30**–**47**) from the genus *Dictyota*.

Sources	Metabolites	Sources/Location	Activities	References
*D. volubilis*	**30**–**33**	Magnetic Island, Queensland, Australia	nd	[25]
	**34**–**41**		nd	[31]
*D. plectens*	9*α*-Hydroxydictyol (**42**) Isodictyol E (**43**)	South China Sea	Antiviral activity	[16]
*D. dichotoma*	Dictyotadiol (**44**)	Patagonia	Weak antifouling activity	[17]
	Dictyohydroperoxide (**45**)	Troitsa Bay, Russian Far East	Moderate cytotoxicity	[37]
	Isopachydictyolal (**46**)	Saronicos gulf, Greece	Antiviral activity	[38]
Genus *Dictyota*	**47**	*Dictyota* spp., Mediterranean Sea	nd	[23]

**Table 3:** Bioactivities of other diterpenes of Group 1 (**48**–**58**) from the genus *Dictyota*.

Structure Class	Metabolites	Sources	Activities	References
Prenylated-germacrane	Hydroxydilophol (**48**)	*D. masonii* Isla Guadalupe, Pacific Mexico	nd	[39]
	Dilophol (**49**)	*D. divaricata*, Great Barrier Reef region	nd	[40]
	3*β*-Hydroxydilophol (**50**)	*Dictyota* sp., Le Brusc Lagoon *D. divaricata*, Great Barrier Reef region	nd	[40,41]
	3*β*-Acetoxydilophol (**51**) Acetoxypachydiol (**52**)	*D. plectens*, South China Sea	Weak antiviral activity	[16]
Prenylated-cadinane	Dictyotins A-C (**53**–**55**)	*D. dichotoma*	nd	[42]
	*Ent*-erogorgiaene (**56**) **57**	*D. dichotoma*, Russian Far-east	nd	[43]
Prenylated-*epi*-elemane	Dictyoxepin (**58**)	*D. volubilis*	nd	[31]

**Table 4:** Bioactivities of dolabellane diterpenes (**59**–**127**) from the genus *Dictyota*.

Sources	Metabolites	Sources/Location	Activities	References
*D. dichotoma*	**59**–**66**	Acicastello, Italy	Antibiotic property	[44]
	**67**	Acicastello, Italy	Strong cytotoxicity	[44,50]
	**68**–**82**	Indian Ocean	nd	[51]
	Dolabellatrienol (**83**)	*D. dichotoma* var. *Implexa*, Red Sea	Moderate cytotoxicity	[15]
*D. pardarlis* f. *pseudohamata*	**84**–**98**	Magnetic Island	nd	[53,54,55]
*D. bartayresiana*	**79**–**82** **98**–**102**	Hare Island, Indian Ocean	nd	[52]
*D. pfaffii*	**103**	Atol das Rocas, Northeast Brazil	Potent antiviral activity; Significant antimalarial activity	[46,57,58]
	**104**	Atol das Rocas, Northeast Brazil	Antifeedant activity; Antiviral activity	[13,46,57]
	**105**	Atol das Rocas, Northeast Brazil	nd	[46]
	Dolabelladienols A - B (**106**, **107**)	Atol das Rocas, Northeast Brazil	Strong antiviral activity	[46]
	Dolabelladienol C (**108**)	Atol das Rocas, Northeast Brazil	nd	[46]
	**109**	Atol das Rocas, Northeast Brazil	Strong anti-HSV-1 activity	[57]
*D. plectens*	**110**–**113**	South China Sea	Specific antiviral activity	[16]
Genus *Dictyota*	**103**	*D. friabilis*, Atol das Rocas reef	Potent anti-HIV-1 activity	[56]
	**114**	*D. divaricata*, Great Barrier Reef region	nd	[40]
	**115**	*D. volubilis*	nd	[31]
	**116**	*Dictyota* sp., near Portopalo	Significant cytotoxicity	[45]
	**117**–**120**	*Dictyota* sp., near Portopalo	nd	[45]
	**121**–**123**	*Dictyota* sp., Le Brusc Lagoon	Antifouling activity	[41]
	**124**, **125**	*Dictyota* spp., Mediterranean coasts, Frence and Algeria	nd	[23]
	**126**	*Dictyota* spp., Mediterranean coasts, Frence and Algeria	Anti-adhesion activity; Antibacterial activity	[23,59]
	**127**	*Dictyota* spp., Mediterranean coasts, Frence and Algeria *D. menstrualis*, Discovery Bay, Jamaica	Anti-adhesion activity; Antibacterial activity; Anti-algal activity	[19,23,41,59]

**Table 5:** Bioactivities of dolastane diterpenes (**128**–**165**) from the genus *Dictyota*.

Sources	Metabolites	Sources/Location	Activities	References
*D. dichotoma*	**128**	Indian Ocean	nd	[51]
	**129** Dichototeraol (**130**) Dichotopentaol (**131**)	Karachi coast, Arabian Sea	nd	[62]
	Dichotenones A and B (**132**, **133**)		nd	[47]
	Amijiol (**134**) Amijiol acetate (**135**) **136**	*D. dichotoma* var. *Implexa*, Red Sea	Antitumor activity; Anti-oxidative activity	[15]
*D. cervicornis*	**137**	Brazil	Strong antimalarial activity; Antifouling activity; Enzyme inhibitory activity	[64,65,66]
	**129** **138**–**140**	Baia da Ribeira, Brazil	nd	[61]
	**141**	Rio de Janeiro, Brazil	Strong antifeedant activity; Antifouling activity; Antiviral activity	[64,66,67,68]
*D. divaricata*	**142**–**145**	Virgin Islands	nd	[69]
*D. indica*	**134** Dictinol (**146**) Dictindiol (**147**) Dictintriol (**148**)	Bulegi, Arabian Sea	nd	[63]
*D. bartayresiana*	**128** **149**–**151**	Hare Island, Indian Ocean	nd	[52]
*D. linearis*	Isoamijiol (**152**) 14-Deoxyamijiol (**153**) Amijidictyol (**154**)		nd	[70,71]
*D. plectens*	**155**–**157**	South China Sea	Weak anti-inflammatory activity	[16]
Genus *Dictyota*	**137**–**138** **158**–**160**	Mixed collections of *D. linearis* and *D. divaricata*, Honduras Bay Islands	nd	[60]
	**161**	Mixed collections of *D. linearis* and *D. divaricata*, Honduras Bay Islands	Strong reversible inhibitory activity	[60]
	**162**	Mixed collections of *D. linearis* and *D. divaricata*, Honduras Bay Islands	Moderate decrease in the twitch height; Weak inhibition of cell division	[60]
	**163**	*D. furcellata*, Cape Peron	nd	[72]
	**164**, **165**	*Dictyota* sp., Canary Islands	nd	[73]

**Table 6:** Bioactivities of secodolastane diterpenes (**166**–**177**) from the genus *Dictyota*.

Structure Class	Metabolites	Sources	Activities	References
Linearols	Linearol (**166**) Isolinearol (**167**)	*D. indica* Arabian Sea *D. cervicornis* Baia da Ribeira Brazil	nd	[74,75]
	Linearol acetate (**168**) Isolinearol acetate (**169**)	*D. cervicornis* Baia da Ribeira Brazil	nd	[74]
Cervicols	Cervicol (**170**) Cervicol acetate (**171**)	*D. cervicornis* Baia da Ribeira Brazil	nd	[74]
Indicols	Indicol (**172**) Indicarol acetate (**173**)	*D. indica* Arabian Sea	nd	[75]
Dichotenols	Dichotenol B (**174**)	*D. dichotoma*	Significant antibacterial and anti-fungal activity	[47]
	Dichotenol C (**175**)	*D. dichotoma*	nd	[47]
Others	Dichotone (**176**)	*D. dichotoma*	Significant antibacterial and anti-fungal activity	[47]
	Dichotodione (**177**)	*D. dichotoma*	nd	[47]

**Table 7:** Bioactivities of xenicane diterpenes (**179**–**218**) from the genus *Dictyota*.

Structure Class	Metabolites	Sources	Activities	References
Monocyclic diterpenes	**179**–**182**	*D. plectens*, South China Sea	Antiviral activity	[16]
	**183**	*D. plectens*, South China Sea	Specific antiviral activity; Strong anti-inflammatory activity	[16]
	**184**	*D. plectens*, Xuwen coast, China	Weak anti-inflammatory activity	[76]
	Acetyldictyolal (**185**)	*D. dichotoma*, Oshoro bay, Hokkaido	High cytotoxicity; Weak antifungal activity	[50,78]
	Hydroxyacetyldictyolal (**186**)	*Dictyota* sp., Le Brusc Lagoon. *D. dichotoma*, Oshoro bay, Hokkaido	nd	[41,78]
	Dictyodial (**187**)	*D. crenulata* *D. flabellata* *D. linearis*, Chios Island	Good antibiotic activity; Antifungal activity	[38,77]
	4*α*-Acetyldictyodial (**188**)	*D. linearis*, Chios Island	nd	[38]
	Hydroxydictyodial (**189**)	*D. spinulosa*, Kin Okinawa	Antibiotic activity; Potent antifeedant	[79]
	**190**	*D. divaricata*, Great Barrier Reef region	nd	[27]
	**191**	*D. ciliolata*, Oualidia lagoon	Moderate antifungal activity	[80]
Bicyclic diterpenes	Dictyotalides A-B (**192**, **193**) Nordictyotalide (**194**) 4-Acetoxydictyolactone (**195**)	*D. dichotoma*, Yagachi Okinawa	Significant cytotoxicity	[12]
	Isodictyohemiacetal (**196**) Dictyodiacetal (**197**)	*D. dichotoma*, Oshoro bay, Hokkaido	nd	[78]
	Dictyolactone (**198**)	*D. dichotoma*	High algicidal activity; Moderate insecticidal activity; Weak antifungal activity; Significant cytotoxicity	[29,50]
	Neodictyolactone (**199**)	*D. linearis*, Chios Island	Weak antifungal activity; Cytotoxicity	[38,50]
	**200**	*D. plectens*, Xuwen coast, China	Antiviral activity; Weak anti-inflammatory activity	[76]
	**201**–**203**	*D. plectens*, Xuwen coast, China	Weak anti-inflammatory activity	[76]
	**204**	*D. plectens*, Xuwen coast, China	Specific antiviral activity; Significant anti-inflammatory activity	[76]
	**205**	*D. plectens*, Xuwen coast, China *Dictyota* sp., Le Brusc Lagoon	Antiviral activity; Weak anti-inflammatory activity	[41,76]
	**206**	*D. plectens*, South China Sea	Weak antiviral activity	[16]
	**207**, **208**	*Dictyota* sp., Bahia de Los Angeles	nd	[81]
	**209**	*Dictyota* sp., Bangsaen Beach, Thailand	Weak anti-tuberculosis activity	[32]
	**210**	*Dictyota* spp., Mediterranean Sea	nd	[23]
	**211**–**213**	*D. divaricata*, Great Barrier Reef region	nd	[27]
Tricyclic diterpenes	**214**	*D. divaricata*, Great Barrier Reef region	nd	[40]
	Ciliolatale (**215**)	*D. ciliolata*, Oualidia lagoon	nd	[80]
	Dictyoepoxide (**216**)	*Dictyota* sp., Bahia de Los Angeles	High vasopressin receptor antagonist activity	[81]
	4*α*-Hydroxycrenulatene (**217**)	*Dictyota* sp., Bangsaen Beach, Thailand	nd	[32]
Bis-diterpene	Dictyotadimer A (**218**)	*Dictyota* sp., Mediterranean Sea	nd	[82]

**Table 8:** Bioactivities of crenulidane, dichotomane, and crenulane diterpenes (**219**–**233**) from the genus *Dictyota*.

Structure Class	Metabolites	Sources	Activities	References
Crenulidanes	Crenulacetal A (**219**)	*D. dichotoma* *D. spinulosa*	nd	[83]
	Crenulacetal B (**220**)	*D. spinulosa*, Yagachi Okinawa	nd	[83]
	Crenulacetal C (**221**)	*D. dichotoma*, Nagahama beach, Ehime	Significant pesticide activity	[84]
	Acetoxycrenulide (**222**)	*Dictyota* spp., Mediterranean Sea *D. dichotoma*, Troitsa Bay, Russian Far East	Weak anti-microfouling activity; Strong fish antifeedant activity	[23,37,83,85]
	**223**	*D. dichotoma*, Troitsa Bay, Russian Far East	nd	[37]
	**224**	*D. divaricata*, Great Barrier Reef region	nd	[27]
	**225**	*D. divaricata*	nd	[40]
	**226**, **227**	*D. plectens*, South China Sea	Weak antiviral activity	[16]
	4*α*-Hydroxypachylactone (**228**)	*D. plectens*, Xuwen coast, China	Moderate anti-inflammatory activity	[76]
	Hydroxycrenulide (**229**)	*Dictyota* sp., Mediterranean Sea	Low antifouling activity	[41]
Dichotomanes	Da-1 (**230**)	*D. menstrualis* *D. pfaffii*, Brazil	Significant anti-HIV-1 activity; Thrombin inhibitor; Antifeedant effect; Inhibitory against pasture weeds	[30,86,87,88,89]
	AcDa-1 (**231**)	*D. menstrualis*, Brazil	Significant anti-HIV-1 activity	[85]
Crenulanes	Sanadaol (**232**)	*D. dichotoma*	High algicidal activity	[29]
	Acetylsanadanol (**233**)	*D. linearis*, Chios Island	nd	[38]
